# Separate units for incarcerated people who committed sexual offenses: luxury or necessity?

**DOI:** 10.3389/fpsyt.2023.1111436

**Published:** 2023-05-25

**Authors:** Elise Wuyts, Minne De Boeck, Tineke Dilliën, Liesbeth Merckx, Kasia Uzieblo, Katrien De Koster, Astrid De Schutter, Kris Goethals

**Affiliations:** ^1^University Forensic Center, Antwerp University Hospital (UZA), Edegem, Belgium; ^2^University Psychiatric Hospital Duffel, Duffel, Belgium; ^3^Collaborative Antwerp Psychiatric Research Institute (CAPRI), University of Antwerp, Antwerp, Belgium; ^4^The Forensic Care Specialists, Van der Hoeven Kliniek, Utrecht, Netherlands; ^5^Faculty of Law and Criminology, Vrije Universiteit Brussel (VUB), Brussels, Belgium

**Keywords:** prison, sex offenders, sex offender treatment, prison climate, policy

## Abstract

**Introduction:**

The policy on treatment of people who commit sexual offenses (PSOs) varies greatly across countries, creating different treatment environments. This study was conducted in Flanders (i.e., the Dutch-speaking part of Belgium) where PSOs receive their treatment in the community. Before this transfer takes place, many PSOs spend time inside prison together with other offenders. This raises the question to what extent PSOs are safe in prison and whether this period would benefit from an integrated therapeutic program. This qualitative research study focuses on the possibility of separate housing for PSOs by examining the current experiences of incarcerated PSOs and contextualizing those with the professional experience of national and international experts in the field.

**Methods:**

Between 1 April 2021 and 31 March 2022, 22 semi-structured interviews and six focus groups took place. Participants were comprised of 9 imprisoned PSOs, 7 international experts on prison-based PSO treatment, 6 prison officer supervisors, 2 prison management delegates, 21 healthcare workers (both inside and outside prison), 6 prison policy coordinators, and 10 psychosocial service staff members.

**Results:**

Nearly all interviewed PSOs reported suffering at the hands of fellow inmates or prison staff because of the nature of their offenses, varying from exclusion and bullying to physical violence. These experiences were corroborated by the Flemish professionals. Consistent with scientific research, the international experts all reported working with incarcerated PSOs who reside in living units separate from other offenders and the therapeutic benefits to this approach. Despite this growing evidence, the Flemish professionals remained reluctant to implement separate living units for PSOs in prisons because of the perceived risk of increased cognitive distortions and further isolation of this already stigmatized group.

**Conclusion:**

The Belgian prison system is not currently organized to create separate living units for PSOs, which has important ramifications for the safety and therapeutic opportunities of these vulnerable prisoners. International experts emphasize a clear benefit for introducing separate living units where a therapeutic environment can be created. Although this would have significant organizational and policy-oriented implications, it would be useful to explore whether these practices could be implemented in Belgian prisons as well.

## Introduction

A significant number of the general population will encounter some form of sexual violence. Keygnaert et al. (1) conducted a large-scale Belgian survey (*n* = 2,115) showing that 64% of respondents were victimized by some kind of sexually transgressive behavior in their lifetime. The study distinguished between hands-off (without touching, e.g., inappropriate sexual proposal) and hands-on (with touching, e.g., sexual assault) sexually transgressive behavior. Here, 78% of women and 41% of men reported having experienced hands-off transgressive behavior. Additionally, 42% of women and 19% of men had ever experienced hands-on transgressive behavior (1). Comparably, data using a more narrow definition of sexually transgressive behavior from the European Union and World Health Organization show that 30% of women will be a victim of sexual violence in their lifetime (2, 3). Among children, an international study from Barth et al. (4) estimates that one in five girls (20%) and slightly fewer than 1 in 10 boys (8%) experience some form of sexually transgressive behavior by the age of 18. Offender-oriented data are more difficult to come by and hypothesized to be a severe underestimation due to the sensitive nature of the offenses and prosecution difficulties (5). The most recent numbers from the Belgian Criminal Justice Department show that 882 people were convicted and 87 people were interned because of rape or assault charges in 2019 (6). The impact of sexual violence is not to be underestimated. This violence is associated with various mental, sexual, and physical health problems for the victim (1, 7, 8) and places an important economic burden on society (9, 10).

People who commit sexual offenses (PSOs) generally present with a heterogeneous profile and they can differ greatly in regard to the nature of their offenses, the underlying motives and potential psychiatric disorders (11–13). Nevertheless, there are therapeutic interventions which have proven to decrease recidivism risk in PSOs as a group and thus contribute to the prevention of sexual violence (14–16). Specialized treatment programs for PSOs are available in most Western, Educated, Industrialized, Rich, and Democratic (WEIRD) countries and either take place within a prison setting (intramural) or outside a prison setting (extramural). Both have shown to have consistent effects on recidivism risk, with Lösel (17) finding a 37% decrease in sexual recidivism, and Gannon et al. (14) finding a similar 32.6% decrease in sexual recidivism. However, intramural therapy programs possess some limitations due to the prison environment. Schmucker and Lösel (15) found in their meta-analysis that extramural PSO treatment generated more consistently positive results compared to intramural treatment, which is attributed in part to the prison climate (18, 19). Prison climate can be defined as the therapeutic and social climate within a prison, including the prison culture and the way in which it works toward decreased recidivism and positive personal change (20, 21). Bosma et al. (22) describe six relevant domains pertaining to prison climate: safety, relationships inside the prison, facilities, contact with the outside world, meaningful activities, and autonomy. Because of the stigma surrounding sexual offenses, PSOs often find themselves low in the prison hierarchy, which impacts the first two domains in particular (23–27). Research has shown that PSOs are often subject to verbal and even physical violence by other inmates and security staff (28–31). Ricciardelli (32) reported that PSOs feel extremely unsafe in the prison environment because of the constant threat of violence. This unsafe environment impacts treatment readiness (33) and engagement in treatment (34). Additionally, negative attitudes toward PSOs among prison staff have been linked to decreased therapeutic alliance and effectiveness, which can impact treatment outcome (35, 36). Because of this precarious situation of PSOs inside prison having safety issues and the subsequent impact on treatment outcome (23, 31, 36), many countries have adopted policies of separate units or even specialized prisons for detained PSOs.

In the United Kingdom (UK), for instance, PSOs fall under the category of vulnerable prisoners and are housed in separate wings inside the prison (32) with the aim of facilitating a safe environment. Similarly, Germany organizes PSO treatment in prison in so-called social-therapeutic units (STUs), separate from other prisoners (37). STUs can be annexed to a regular prison, but may also function independently as housing for vulnerable prisoners. In the case of separate units, the risk of incidents remains because these separate wings are still part of the prison as a whole, which results in occasional contact between PSOs and other offenders (34). To address these concerns, the UK has recently set up several specialized PSO prisons (36). Recent studies on these prisons show that a stay here has positive effects on the mental health of the PSOs and therefore on their therapeutic opportunities (38). Blagden and Perrin (34) conducted research on the experiences and perspectives of PSOs (*n* = 16) and prison staff (*n* = 16) within one of those specialized prisons. The researchers concluded that the prison climate was generally described as positive and conducive to the rehabilitation of the PSOs, both by prison staff and prisoners. The PSOs themselves also reported feeling safer, which facilitated personal growth. Both parties conveyed a positive and constructive interpersonal relationship (34).

In Flanders (i.e., the Dutch-speaking part of Belgium), PSO treatment mainly occurs in an extramural setting, mapped out in a specific policy framework where different governments are responsible for the management and treatment of PSOs. The Flemish government is responsible for healthcare and for the implementation of treatment for PSOs, and the Federal government is responsible for the juridical follow-up of PSOs (39). Many PSOs first spend time in prison before they are transferred to an inpatient or outpatient treatment setting where they receive specialized treatment (i.e., treatment specifically focused on recidivism risk reduction in PSOs). Before this transfer takes place, the PSOs spend time with other offenders while incarcerated. This raises the question to what extent PSOs are sufficiently safe in prison and whether this period would benefit from an integrated therapeutic program in preparation of the treatment offered in the community.

This qualitative study focuses on the environment in which such a therapeutic program might be embedded by examining the current experiences of incarcerated PSOs and contextualizing those with the professional experience of several national and international experts in the field. This study further explores the option of separate living units inside Flemish prisons to facilitate the implementation of specialized treatment for PSOs in prison. These results will be used to make recommendations for improving the circumstances of imprisoned PSOs in Flanders with the aim of providing an environment in which specialized PSO treatment can be offered.

## Materials and methods

This study was conducted between 1 April 2021 and 31 March 2022 as part of a larger research project concerning the current situation of incarcerated PSOs and intramural treatment options. It was financed by the Flemish minister for Justice and Enforcement and approved by the ethical committee of the Antwerp University Hospital (UZA). All collected data were stored in accordance with General Data Protection Regulation (GDPR) legislation on secure cloud servers of the respective partners (UZA and VUB) with data transfer agreements for every information exchange. The data were further pseudonymized throughout the process to meet the principles of confidentiality and integrity.

A qualitative methodology was chosen to allow for the collection of detailed and context-sensitive information (40), using semi-structured interviews and focus groups. A semi-structured topic list was prepared in advance based on the literature study done beforehand to ensure all topics were addressed, while still leaving room for the interviewer or focus group leader to explore certain topics more deeply throughout the process (40, 41). All interviews and focus groups were conducted by the first and seventh author, both educated and experienced in the research methods used here. Focus groups are considered a superior method of data collection because they facilitate an exchange of thoughts between respondents that provides researchers with growing insight in an area of research through the meaning attached (42). Moreover, focus groups provide an opportunity for validating the social dynamic between respondents and offers more space to react to the opinions of others. This method is therefore considered a more ‘natural’ way of data collection (42). Single or double interviews were conducted with participants where a group format was not possible because of organizational issues. Several interviews had to be conducted online, either because of the location of the international experts, or because of quarantine measures imposed by the COVID-19 pandemic. All respondents were provided with a detailed letter and an informed consent form which they signed before the interview or focus group was conducted.

### Interviews with incarcerated PSOs

Based on previous research charting the number of PSOs inside Belgian prisons (43), six Flemish prisons were chosen for inclusion as they housed relatively large numbers of PSOs. These prisons are located in Hoogstraten, Beveren, Leuven, Vorst, Bruges, and Merksplas. Coordinators of psychosocial services in these prisons acted as intermediaries and discretely approached PSOs to assess whether they were willing to participate in this study. Nine PSOs were included in the study. All necessary steps were taken to ensure the anonymity of the PSOs with the help of the intermediaries and by using discretion when approaching the PSOs for interviewing.

### Interviews with prison officer supervisors

Since previous research has shown that interviews with prison officers often generate very heterogeneous results (44), the choice was made to approach the direct supervisors of the prison officers instead. All supervisors active in the six prisons mentioned above were contacted through the general prison directorate to assess whether they were interested in participating. Six prison officer supervisors were included in the study.

### Interviews with delegates from the general prison directorate

Because of their helicopter view on prison policy, two management delegates were included in this study. They were directly contacted by the researchers to assess whether they were interested in participating.

### (Duo)interviews with international experts

Several international experts were identified through targeted sampling and invited to participate in an online interview. Experts were identified as authorities in this particular research field based on their publications and professional involvement in providing specialized PSO treatment in a prison setting. The seven included experts represented the following countries: United Kingdom (*n* = 3), Sweden (*n* = 1), North America (*n* = 2), and the Netherlands (*n* = 1).

### Focus groups with healthcare workers, prison policy coordinators, and psychosocial service staff

In total, six focus groups were conducted: four focus groups consisting of both healthcare workers (*n* = 21) and prison policy coordinators (*n* = 6), and two focus groups consisting of psychosocial service staff (*n* = 10). Healthcare workers (providing specialized or general treatment for PSOs) were included from organizations currently providing treatment for PSOs, both inside and outside of prison. Overall, 24 organizations were identified and contacted, 19 of which agreed to participate in the focus groups. Prison policy coordinators, present in all Flemish prisons, survey all services pertaining to healthcare and wellbeing of the prisoners. All these coordinators, as well as the psychosocial service staff members (responsible for psychosocial advice and assessment in prison), were contacted through the general prison directorate to assess whether they were interested in participating.

### Data analysis

All qualitative data were thematically analyzed using the qualitative analysis program NVIVO (45). In a thematic analysis, different categories are established and systemically linked (46). This allows detailed and varied information to arise from the qualitative data. This type of analysis is especially useful when the focus of the research is on the opinions, knowledge, and vision of certain people. A thematic analysis discerns itself from other qualitative data analysis methods because it is not bound to specific theoretical prepositions (46).

The interview and focus group transcriptions were coded using a directional coding scheme where different codes can be accommodated (45). To increase interrater reliability and research validity, all transcriptions were coded by both researchers, the first and seventh author, firstly independent from each other (47) and secondly in collaboration to develop a codebook that both researchers were in agreement on. All codes and subsequent final themes can be found in Table 1. It is important to note that for the scope of this article, only the following themes were included in the analysis: “prison structure”; “relationships between PSOs and other inmates”; “relationships between PSOs and prison officers.” These themes were chosen to adequately answer the research question of this study.

**TABLE 1:** List of final themes and related specific codes.

Final themes	Specific codes
**Prison climate**	
Prison infrastructure	• Facilities • Separate housing
Relationships between PSOs and other inmates	• Personal safety • Stigma • Isolation • Loss of anonymity
Relationships between PSOs and prison officers	• Dynamic safety procedure • “Warm” and “cold” prison officer structures • Prison officer training
Relationships between prison officers and healthcare workers	• Collaboration between Flemish and Federal government • Therapeutic confidentiality
Therapeutic climate	• PSO wellbeing • Healthy sexuality • Treatment readiness • Autonomy
**Treatment programs**	
General treatment	• Pre-therapeutic interventions • Collaboration with extramural treatment programs • Treatment continuity • Reintegration
Specialized treatment	• Group therapy versus individual therapy • Voluntary treatment versus mandatory treatment
**PSO**	
PSO profile	• Therapeutic willingness • Treatment goals
Denial	• Partial denial • Full denial

The final themes and codes abovementioned represent the data analysis of the research project as a whole. The themes discussed in this study are only a part of this.

Afterward, all respondents were presented with the written results to provide feedback if necessary. The cited quotes used in the results serve as a means of supporting and clarifying these viewpoints. It should be noted that except for the interviews that were conducted with the international experts, all interviews and focus groups were conducted in Dutch. The following quotes are therefore translated by the researchers and do not constitute verbatim speech from the respondents.

## Results

The following sections give an overview of the different viewpoints of the respondents included in this study. Firstly, the results from the PSO interviews are addressed. Secondly, all Flemish professional respondents are discussed as a group, which includes the healthcare workers, prison officer supervisors, general prison directorate delegates, prison policy coordinators, and psychosocial service staff. Last are those from the international expert interviews. The results are further divided according to the theme discussed. Relevant to this study, these themes pertain to the safety of PSOs in prison and the discussion around separate housing for PSOs in prison. Only the international experts did not discuss the safety of PSOs in prison as a separate theme.

### People who committed sexual offenses

#### Safety of PSOs in prison

The PSOs reported widely varied experiences with their fellow inmates. For instance, they emphasized the impact of a good cellmate on the way they experienced their incarceration. However, the included PSOs also mentioned the stigma on sexual offenses that lives inside the prison setting and the shame they experienced with respect to their own offenses.

*I can’t quite believe that I’m also guilty of these kinds of offenses. It’s something that gnaws at me, that I stooped to that. I’d rather be arrested for dealing drugs [*…*] than being here for these kinds of offenses. I’m ashamed of it (PSO 5, 10/11/2021).*

According to the respondents, their shame was amplified by the hostile attitudes of other inmates toward PSOs. Almost all of them indicated having to be constantly on guard, as they experienced an increased risk of verbal or physical violence. Several respondents reported victimization at the hands of their fellow inmates. One respondent also indicated having been victim to sexually transgressive behavior from another inmate. Most PSOs reported that they were afraid of finding themselves in dangerous situations on a daily basis.

*So actually, I’m just walking around all day afraid that they’ll push me into a corner somewhere and I won’t come out alive. It’s as simple as that (PSO 2, 5/11/2021).*

The respondents also commented on the strategies they employed to ensure their personal safety as much as possible inside prison. An important aspect of this was to keep the true nature of their offenses hidden, which resulted in a constant fear of being discovered.

*As long as they don’t know why you’re there, it’s all right. You just make up some kind of story that’s reasonably believable. You have to know of course what story you’re telling, and don’t make up some sort of crazy story because then they’ll know something’s wrong. So, you have to come up with something credible and then they’ll leave you alone (PSO 2, 5/11/2021).*

A few of the respondents had already lost this anonymity and were forced to isolate themselves from the general prison population. These PSOs reported that they never left their cell out of fear of being harassed. For instance, they expressed the necessity of separate leisure time moments or separate transfers to and from visiting rooms and workplaces.

With respect to the prison officers, the PSOs reported widely varied experiences. Some respondents talked about having a good relationship with prison officers that was based on mutual respect, whereas others reported feeling unsafe because of actions of the prison officers, especially when it came to safeguarding their anonymity. Several reported that as soon as prison officers found out about the sexual nature of their offenses, this information seemed to spread very quickly to the rest of the prison population. Moreover, respondents noted not always being treated equally by prison officers when the nature of their offenses was revealed. This ranged from receiving a “cold shoulder” to inadequate interventions when violence was perpetrated against them.

*Often when the prison officer knows, he will tell other inmates who will then force something. And also, if the prison officer knows and something happens, he will turn his head, like he hasn’t seen it. And then the person who did something won’t get punished. Because the officer didn’t see it. And in here, if the officer didn’t see, you can’t get punished (PSO 2, 5/11/2021).*

#### Separate housing for PSOs in prison

All PSOs except one reported a preference for being housed in a separate unit or a specialized prison. The most important reason they gave for this was not having to live in fear of being discovered by their fellow inmates or the prison officers. It would increase their feeling of safety and would create more openness toward specialized therapeutic interventions.

*I’d say yes. I’d say yes straight away. Absolutely. I wouldn’t have to hide anymore (PSO 2, 5/11/2021).*

*Flemish professionals (i.e., healthcare workers, prison officers supervisors, general prison directorate delegates, prison policy coordinators, and psychosocial service staff).*

#### Safety of PSOs in prison

All respondents had been confronted with the precarious situation of working with incarcerated PSOs and their low hierarchal position. According to them, PSOs were often the victim of bullying and both verbal and physical violence by their fellow inmates. The prison officer supervisors in particular noted that these issues probably occurred much more frequently than the incidents reported to them. Several prison officer supervisors, however, stated that physical violence was less common than verbal aggression. They speculated that this was due to the zero-tolerance policy toward physical violence that existed in many prisons and the possible consequences linked to that, such as loss of privileges or transfer from open to closed regime.

The respondents also reported that PSOs used different strategies to deal with their vulnerable status. Many of them chose to lie about the nature of their offenses in order to blend in with the general prison population. Those for whom lying was not an option, would often isolate themselves from the other inmates, or group together with other PSOs. The healthcare workers also indicated that other inmates often use the image of “the typical sex offender,” based on stereotypes and stigmatizing attitudes, to unmask PSOs who are trying to remain anonymous.

*What we also see is that certain sex offenders will find each other, if they’re out in the open they have a certain connection. They’ll play a board game during leisure time, for instance, and then you immediately get that reputation of “ah, that’s the rape group” (focus group healthcare workers and policy coordinators, 10/09/2021).*

When it came to guaranteeing the safety of the incarcerated PSOs, the prison officers had an important role to play. Although all respondents agreed that the majority of the prison officers interacted with PSOs in a professional manner, they did remark that sometimes this was not the case. The prison officer supervisors confirmed that some prison officers experienced aversion or prejudice toward PSOs, which could lead to incidents.

*It happens from time to time that we must correct employees because we see that they can’t separate the perception they have of child molesters, which is of course negative in many people, especially people who have children of their own, and then you notice they take those perceptions with them. Which isn’t professional and with which we don’t agree of course, so we step in (prison officer supervisor 2, 17/09/2021).*

According to the healthcare workers, these prejudices against PSOs were a reflection of the predominantly negative perception regarding PSOs in society. They added that they saw a pressing need for increasing awareness and educating society in general but also prison officers specifically. They saw opportunities in paying closer attention to vulnerable prisoner groups such as PSOs during the basic training of prison officers for instance.

#### Separate housing for PSOs in prison

The question of housing PSOs in a separate prison or prison unit brought forth a multi-faceted discussion among the professional stakeholders included in this study. There was consensus on the positive effects this might have on the safety of PSOs, but respondents were worried about possible negative ramifications as well. Several commented that housing PSOs separately will inevitably result in the loss of their anonymity, which may lead to an even stronger stigmatization among other detainees. The prison officer supervisors acknowledged this and argued that all other aspects of prison life should be organized separately as well. Since the PSOs will lose their anonymity, activities such as leisure time, work, lessons, and religious services must then take place separate from the general prison population to ensure their safety.

Another issue raised among respondents was the possibility that placing PSOs together as a group might lead to a reinforcement of cognitive distortions surrounding their sexual offenses. The extramural healthcare workers in particular advocated for mixed offender living situations to stimulate reintegration because this reflects the reality outside of prison.

*I think there’s people who’d prefer that purely out of safety concerns. But I think if you look at the bigger picture, it’s not so ideal because you’re affirmed in “you’re a sex offender and you can’t integrate with those other people.” You won’t be accepted otherwise and that seems to me like it will increase the risk if someone goes back to society. If you’re going to feel that stigma so strongly, that doesn’t seem ideal (focus group healthcare workers and policy coordinators, 16/09/2021).*

*You offer safety to those people and that aspect is very closely linked to trust. And trust and self-confidence are often already difficult with them. So, I think that you can only take steps with these kinds of people when you create a safe environment for them. If you place them together, I think that environment is safer than when you place them with the rest (prison officer supervisor 2, 17/09/2021).*

The general prison directorate delegates built upon the idea of separate housing by suggesting to tailor the available services to those units or prisons as well. This would entail that different treatment programs were offered in different prisons, and incarcerated PSOs were placed in a prison suited to their specific treatment needs. This idea was supported by other respondents as well.

*The way I see it, that way you can have a treatment program fitted to that prison. I would want people to be sent in a deliberate way, like “okay, in that prison there’s this and this and this, you seem like you might fit there. Maybe you could go there” (focus group healthcare workers and policy coordinators, 16/09/2021).*

However, there were some critical voices as well. Firstly, the participants acknowledged the problem of overpopulation that has long been an issue in Belgian prisons. This means that often, prisoners are simply placed where there is still room available. Secondly, their place of residence should also play an important role in their placement. This is crucial to ensure that friends and family have the means to visit during the imprisonment and to promote a safe reintegration into the community.

### International experts

#### Separate housing for PSOs in prison

All respondents agreed on the benefits of housing PSOs separately from the other inmates. A separate unit or prison, they argued, not only improves their physical safety, but also the emotional safety needed for a therapeutic process. One respondent in particular emphasized this creates a more therapeutic environment, which offers more opportunities for a treatment program as opposed to the generally more oppressive climate inside a prison.

*So, they all live together within one living unit. The staff can be more focused on them, they interact with each other as well and they can support each other to some extent. It’s easier to control the environment and make it more therapeutic rather than the other way around (international expert 3, 12/10/2021).*

When asked to comment on the issues raised by the Flemish professional respondents about the reinforcement of cognitive distortions and increased stigmatization, the international experts emphasized that there is no scientific evidence to corroborate these concerns. In their view, these concerns arise from fear or a “gut feeling,” and they warned against letting this influence policy making. According to them, these perceived issues do not pose a hurdle toward implementing separate housing for PSOs in prison, though the experts did stress the importance of embedding a therapeutic environment within the separate unit or prison. Building on that, they stated that this should also entail specialized training for all staff involved, specifically the prison officers who work with PSOs.

## Discussion

Almost all PSOs included in the current study reported feeling unsafe in prison in one way or another, which was also confirmed by the Flemish professional respondents. This feeling of being unsafe is strongly linked to the precarious situation of PSOs in prison, where they find themselves at the bottom of the hierarchal ladder. Those PSOs who can successfully conceal the nature of their offenses sometimes succeed in blending with the general prison population, but they live in fear of being discovered. Lying about their offenses, however, is not a realistic coping strategy for all PSOs. International research indicates that incarcerated PSOs who are unable to maintain their anonymity inside the prison setting are under constant threat of verbal, physical, and even sexual violence (30–32).

In addition to the threats posed by fellow inmates, PSOs sometimes encounter problems from prison officers as well. Several PSOs included in this study indicated a strained or even harmful relationship with prison officers. This is confirmed by the Flemish professional respondents who often noticed an ambiguous or even hostile attitude from some prison officers toward PSOs. Some prison officers do not respect the anonymity of the PSOs, even failing to act when other inmates put PSOs in danger. International research also supports these claims of maltreatment by both other inmates and prison officers (28–31). Because of these important safety concerns, other countries such as the UK and Germany house PSOs in separate prisons or prison units (34, 37, 48). Nearly all PSOs in this study stated they would prefer a similar setting to the one they are in now. To them, a separate section or specialized prison implied an increased sense of safety, both physically and mentally. They would not have to live in constant fear that their offenses might become public knowledge.

However, several issues were raised by professional respondents regarding the practical application of separate housing. The concerns of the Flemish professional respondents that placing PSOs together as a group might lead to a reinforcement of cognitive distortions or might complicate their reintegration process is not corroborated by empirical evidence (38) or by the international experts interviewed in this study. The international experts did argue that this underlined the importance of incorporating treatment in a separate housing system. By having a therapeutic framework, these issues could be addressed constructively as they arise. Another important issue raised by the prison officer supervisors in this context, which is confirmed by studies on the separate vulnerable prisoner units in the UK (32), is the importance of providing not only separate housing, but also separate lessons, work, leisure time, and religious services. Such activities should be organized separately from the prison population given that PSOs are more visible as a group when housed separately. Lastly, the respondents stressed that consideration should be given to the training of prison officers who work with PSOs. The prison officer supervisors supported this idea of increasing awareness of vulnerable prisoner groups such as PSOs in the basic training and further education of prison officers. Research also confirms the importance of training personnel that regularly come into contact with PSOs (31, 49), showing that prison officers who have more knowledge on and experience in dealing with PSOs generally have a more positive attitude toward them (50, 51).

As mentioned by the international experts, separate housing would also provide more therapeutic opportunities. Blagden (20) shows that the prison climate needs to be conducive to implementation of treatment programs for them to be successful. A supportive prison climate has been seen to facilitate treatment willingness and is correlated with the belief amongst prison staff that PSOs can change their behavior (20, 52, 53). Moreover, a safe environment facilitates treatment readiness (33) and engagement in treatment among PSOs (34). Additionally, positive attitudes from staff toward PSOs influence the therapeutic alliance and effectiveness of therapeutic interventions (35, 36). The included PSOs also reported finding support amongst themselves in the prison setting. The international literature confirms the important role fellow inmates play in the participation and retention of PSOs in treatment (20). When considering the possibility of implementing specialized PSO treatment in Flemish prisons, separate living units have important implications with respect to treatment outcomes and recidivism risk. The international experts likewise argued that this separation is necessary to create a therapeutic environment where a treatment program can be embedded. The Flemish professionals acknowledged that this kind of arrangement would positively impact both the prison climate and the treatment opportunities for PSOs. This is further corroborated by international research, which reports that both the PSOs and the prison staff have a more positive attitude within PSO-specific prisons with both describing their relationship as more positive and constructive than in a general prison setting (34, 38).

### Policy implications

This research was commissioned by the Flemish government to formulate several recommendations regarding the situation of PSOs in prisons and the possibility of establishing an intramural treatment program. Based on the results of this study, the researchers put forth the recommendation to provide housing for incarcerated PSOs separate from the general prison population to better guarantee their safety. There is an evidence base for this design, both as a separate unit and as a specialized prison (34, 37, 48) and these initiatives provide a more therapeutic prison climate that is beneficial to creating a safe environment suited to implement treatment programs (34, 38).

Because of concerns from the Flemish professionals regarding accessibility of the prisons and the importance of visits from friends and family, the implementation of specific units for PSOs inside general prisons is preferred over a specialized prison for PSOs. Establishing smaller, separate sections within current prison settings allows a wider geographical spread compared to centralizing a large group of PSOs in a single Flemish prison. This promotes the maintenance of social relations when incarcerated, which reduces recidivism risk (54–56). This choice does have implications for the organization of daily life inside the prison, such as movements to and from workplaces, lessons, and leisure time. These should all be organized strictly separate from the general prison population as well, given that the PSOs are more visible as a group when they are housed separately. Special attention should also be given to increasing awareness and training of the prison officers who encounter PSOs. Separate housing also allows more specific placement of prison officers with positive attitudes toward PSOs. These prison officers should receive training where special consideration should be given to vulnerable prisoner groups such as PSOs in order to develop these positive attitudes. Lastly, separate housing allows for establishing a therapeutic environment within the separate PSO unit where a treatment program can be implemented for all incarcerated PSOs.

Because the current Flemish prison system is not yet organized to provide separate housing nor specialized treatment programs for PSOs, these recommendations will need to be carefully translated to practice. A pilot project would facilitate this process by implementing a separate unit inside a prison best suited for this infrastructure. This may provide a steppingstone to further implementation, which creates opportunities for positively impacting the circumstances of incarcerated PSOs, the treatment of PSOs, and ultimately on the prevention of sexual violence.

The following recommendations can therefore be distilled from the current research:

- Establishment of separate units for PSOs in Flemish prisons starting with a pilot project
- Careful selection, training, and further education of prison officers
- Integration of a specialized therapeutic program.

### Limitations

All research is subject to limitations, several of which should be mentioned here. Firstly, there is a possible selection bias in the recruitment of respondents. Specifically PSO recruitment may have been affected because the psychosocial service staff was asked to act as an intermediary in the selection process. Additionally, several recruitments were conducted through the general prison directorate, which also increases the possibility of selection bias if, for instance, the directorate is not up to date on its employee status. Secondly, because several interviews had to be conducted online, this may have led to decreased non-verbal communication (57). Thirdly, the original intent of organizing online focus groups with international experts had to be reworked to (duo)interviews because of several last-minute cancelations. These interviews still generated the information needed for this research but lacked the dynamic component characteristic of focus groups. Lastly, it should be noted that qualitative data has a limited generalizability potential because of the generally small sample size, though that is not the aim of qualitative research.

## Conclusion

The current Belgian prison system does not provide separate living units for PSOs, which has important ramifications for the safety and wellbeing of these vulnerable prisoners, as observed by the included PSOs and Flemish professionals as well as confirmed in scientific research. Results of the current study are in line with the empirical evidence showing important benefits to separate housing for PSOs. These initiatives provide a safer and more therapeutic prison climate that is beneficial to the implementation of treatment programs. Based on this research, separate housing for incarcerated PSOs can be recommended for implementation in Flanders. Although this would have significant organizational and policy-oriented implications, it would be useful to explore how this could be implemented in current Flemish policy inside the Belgium prison system.

## Data availability statement

The datasets presented in this article are not readily available because ensurance of anonymity. Requests to access the datasets should be directed to EW, elise.wuyts@hotmail.com.

## Ethics statement

The studies involving human participants were reviewed and approved by Ethical Committee of the Antwerp University Hospital. The patients/participants provided their written informed consent to participate in this study.

## Author contributions

EW and ADS conducted the research under supervision of MDB, TD, LM, KG, KU, and KDK. EW wrote the article with feedback from MDB, TD, LM, KG, KU, and KDK. All authors contributed to the article and approved the submitted version.
